# Capacity Degradation of Zero-Excess All-Solid-State Li Metal Batteries Using a Poly(ethylene oxide) Based Solid Electrolyte

**DOI:** 10.1021/acsami.4c03387

**Published:** 2024-06-12

**Authors:** Philipp Müller, Conrad Szczuka, Chih-Long Tsai, Sandro Schöner, Anna Windmüller, Shicheng Yu, Dominik Steinle, Hermann Tempel, Dominic Bresser, Hans Kungl, Rüdiger-A. Eichel

**Affiliations:** † Institut für Energie- und Klimaforschung (IEK-9: Grundlagen der Elektrochemie), Forschungszentrum Jülich, Jülich 52425, Germany; ‡ Institut für Materialien und Prozesse für elektrochemische Energiespeicher- und wandler, RWTH Aachen University, Aachen 52074, Germany; § Helmholtz Institute Ulm (HIU), Ulm 89081, Germany; ∥ Karlsruhe Institute of Technology (KIT), Karlsruhe 76021, Germany; ⊥ Institut für Energie- und Klimaforschung (IEK-12: Helmholtz-Institute Münster, Ionics in Energy Storage), Forschungszentrum Jülich, Münster 48149, Germany

**Keywords:** polymer electrolyte, anode-free, zero-excess, PEO, Li anode, interface

## Abstract

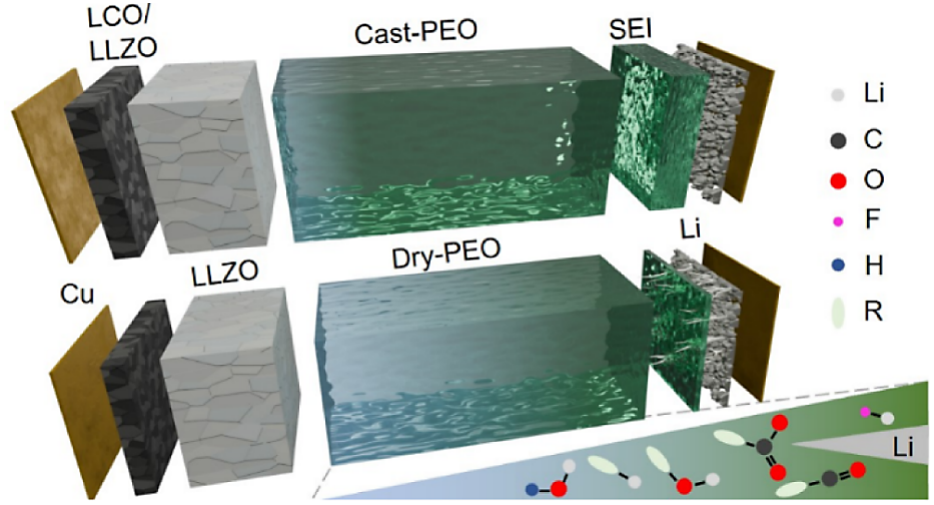

Solid-state polymer electrolytes (SPEs), such as poly(ethylene oxide) (PEO), have good flexibility when compared to ceramic-type solid electrolytes. Therefore, it could be an ideal solid electrolyte for zero-excess all-solid-state Li metal battery (ZESSLB), also known as anode-free all-solid-state Li battery, development by offering better contact to the Cu current collector. However, the low Coulombic efficiencies observed from polymer type solid-state Li batteries (SSLBs) raise the concern that PEO may consume the limited amount of Li in ZESSLB to fail the system. Here, we designed ZESSLBs by using all-ceramic half-cells and an extra PEO electrolyte interlayer to study the reactivity between PEO and freshly deposited Li under a real battery operating conduction. By shuttling active Li back from the anode to the cathode, the PEO SPEs can be separated from the ZESSLBs for experimental studies without the influence from cathode materials or possible contamination from the usage of Li foil as the anode. Electrochemical cycling of ZESSLBs shows that the capacities of ZESSLBs with solvent-free and solvent-casted PEO SPEs significantly degraded compared to the ones with Li metal as the anode for the all-solid-state Li batteries. The fast capacity degradation of ZESSLBs using different types of PEO SPEs is evidenced to be associated with Li reacting with PEO, residual solvent, and water in PEO and dead Li formation upon the presence or absence of residual solvent. The results suggest that avoiding direct contact between the PEO electrolyte and deposited lithium is necessary when there is only a limited amount of Li available in ZESSLBs.

## Introduction

Lithium-ion batteries (LIBs) are nowadays one of the key energy storage systems, enabling portable electronic devices and electric vehicles, and being increasingly used also for short-term grid electrical storage.^1−3^ Much research has been conducted on how to improve this technology, creating batteries higher in energy density, cheaper in price, and safer in operation.^4−6^ Solid-state electrolytes, which have higher thermal and mechanical stability compared to state-of-the-art organic liquid electrolytes (LE) used in LIBs, have therefore caught the attention of the research community. It is thought that the higher mechanical stability could hinder the growth of Li dendrites, higher thermal stability could alleviate the thermal runaway problems, and higher electrochemical stability could suppress Li loss from continuous formation of the solid electrolyte interphase (SEI).^7−9^

PEO SPE was first introduced in 1973 by Fenton et al.^10^ This SPE can be formed by dissolving alkali salts in PEO, which later opened the field of using polymers as solid electrolytes for electrochemical devices. Nowadays, PEO is considered as one of the most promising SPE for solid-state Li battery development due to its environmental friendliness, low cost, high flexibility, low weight, and easy processability. However, Li batteries using PEO as the solid electrolyte are usually reported with low Coulombic efficiencies.^11−16^ One of the identified reasons for the low Coulombic efficiencies is attributed to the PEO decomposition when paired with a cathodic working voltage higher than 4.0 V vs Li/Li^+^.^15,17,18^ On the other hand, Sahore et al. demonstrated that the Li metal loading in Li/PEO/Li symmetric cells can dramatically affect the Coulombic efficiencies of the cells, which indicates the possible instability between PEO SPE and metallic Li.^19^ To compensate for the issue of Li loss during cycling with PEO SPE, an excessive Li reservoir is necessary. An explanation is the low mechanical stability of PEO cannot impede dendrite formation, which subsequently leads to electrochemically inactive “dead Li” formation.^16,20−22^ Other research has attributed this instability to different reasons, such as residual casting solvents or trace water introduced during the manufacturing processes that reacts with Li on the anode side.^23−26^ Although PEO was long thought to be electrochemically stable against a Li anode,^27^ more recent investigations call PEOs’ stability toward Li into question. In difference to earlier Ab initio molecular dynamics (AIMD) modeling by Ebadi et al., which predicted no reaction between Li and PEO,^28^ Ushakova et al. show energetic preferences for bond cleavage in PEO when in contact with Li by using density functional theory modeling,^29^ while Mirsakiyeva et al. suggest the formation of insulating Li_2_O in the case of PEO decomposition.^30^ Also, AIMD modeling by Wu et al. pointed out that single Li atoms, as well as nondefect free Li surfaces, exhibit higher reactivities for PEO bond cleavage by Li·^31^ According to their AIMD model, the contact between PEO and Li results in the formation of lithium alkoxides, ethylene, and Li_2_C_2_H_4_. Although these decomposition products were detected by XPS, the authors of these publications carried out their experiments by contacting metallic Li to only solvent-casted PEO films, possibly enabling side reactions through activation by solvent–Li reactions.^29,32^ To our knowledge, there is no characterization of PEO–Li interphases, which were subjected to the full cell electrochemical cycling conditions, especially when considering a long-term environment under the high electric field across the anode/electrolyte interface, and the Li stripping/deposition may have a high impact on the stability of PEO toward metallic Li.

This study is thus trying to expand the insight into the PEO–Li interface/interphase evolution during electrochemical cycling of a full battery in combination with exploring the possibility of using PEO as the solid electrolyte for developing zero-excess all-solid-state Li metal batteries (ZESSLBs). To also address the previous concerns of side reactions initiated by residual solvents, solvent-free and acetonitrile (ACN)-casted PEO were fabricated for comparison. To avoid PEO degradation from direct contact with the cathode active material, ceramic half-cells made of Li_6.45_Al_0.05_La_3_Zr_1.6_Ta_0.4_O_12_ (LLZO) as the solid electrolyte and LiCoO_2_/LLZO as the composite cathode were used as platforms to study the effect of using an extra PEO solid electrolyte layer on the battery cells performance. The ZESSLB concept with a ceramic half-cell also made it possible to separate the PEO SPEs from the rest of the battery components by fully discharging the cell to shuttle all active Li back to the cathode. As the Li is only plated onto the current collector during charge and can be potentially completely removed upon discharge, all side reaction products on the PEO can be expected to be originated from the reactions between the deposited Li and PEO, which reduces the possibility of misinterpreting sputter damage or contamination from used Li foils.^29,33^ Additionally, this method allows for the investigation of a long-term cycled PEO–Li interphase instead of just the equilibrium PEO–Li interface. A more realistic PEO–Li interphase, including possible electrochemical degradation products, can therefore be investigated.

## Results and Discussion

**Figure 1:**
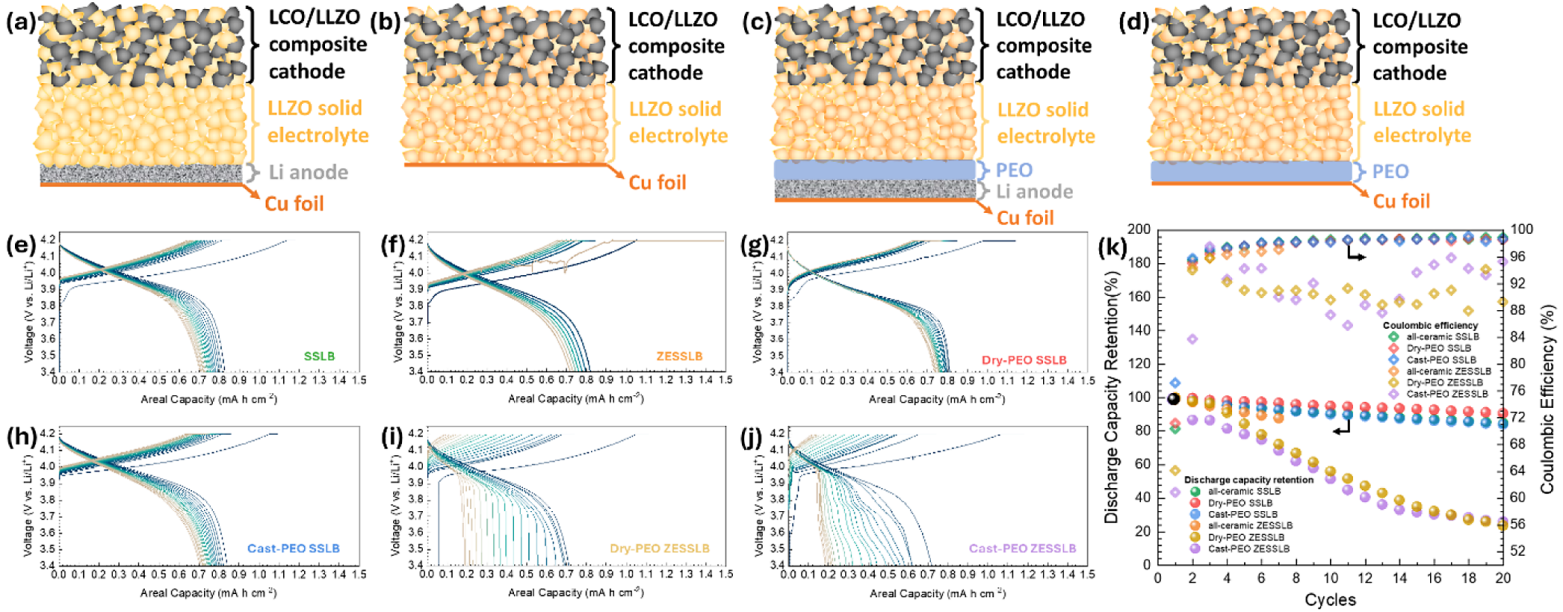
Sketch of different types of prepared battery cells, (a) SSLB, (b) ZESSLB, (c) SSLB with PEO SPE, and (d) ZESSLB with PEO SPE. The corresponding electrochemical cycle performances of different types of battery cells, (e) SSLB, (f) ZESSLB, (g) dry-PEO SSLB, (h) cast-PEO SSLB, (i) dry-PEO ZESSLB, (j) cast-PEO ZESSLB, and (k) their capacity retentions and Coulombic efficiencies.

To ensure the functionality of the ceramic half-cells, two types of SSLB cells using only the LLZO electrolyte were manufactured. First, a cell was assembled by attaching Li foil onto the LLZO to form the SSLB, Figure 1a. The cell was electrochemically cycled with a current density of 50 μA cm^–2^ between 4.2 and 3.4 V vs Li/Li^+^ at 80 °C, Figure 1e. The charge/discharge curves show that the assembled battery performed as a typical SSLB by using LLZO as the solid electrolyte and LCO/LLZO as the composite cathode. The cell capacity fading was understood to be caused mainly by interfacial delamination between LLZO and LCO owing to the stress evolvement during electrochemical cycling, microcrack formation in LCO, and partial decomposition of LCO into metallic Co from LCO lattice oxygen deficiency.^34^ The ZESSLB was made by attaching Cu foil directly to the LLZO solid electrolyte of the ceramic half-cell, Figure 1b. Its electrochemical charge–discharge performance is similar to that of the SSLB, Figure 1f. However, an extended charging process accompanied by voltage noise was observed at the eighth charging cycle due to Li dendrite formation. This should be a result of inhomogeneous Li deposition onto the Cu foil caused by the lithiophobic nature of Cu as well as inhomogeneous Li deposition currents due to uneven contact between the rigid Cu and LLZO layers.

To study the compatibility between PEO and Li, two further types of cells were assembled by attaching PEO SPEs onto the LLZO side of the ceramic half-cells; SSLBs by using Li foil as the anode, Figure 1c, and ZESSLBs using only Cu foil as the current collector on the anode side, Figure 1d. Two types of PEO SPEs, i.e., solvent-free PEO (dry-PEO) and ACN-casted PEO (cast-PEO), were prepared to study the impact from different SPE preparation methods onto their electrochemical performances. Figure 1g,h shows the electrochemical cycling performances of dry-PEO and cast-PEO SSLBs, respectively. The electrochemical performance of the cast-PEO SSLB is similar to that of the SSLB without PEO SPE, while the dry-PEO SSLB gave a small improvement in the cycling performance compared to the other two. The capacity retentions of the SSLB and cast-PEO SSLB were thus about 84% after 20 cycles while the dry-PEO SSLB retained about 90% of its initial capacity, Figure 1k. The enhanced performance for dry-PEO SSLB can be attributed to a lower ohmic resistance increase during cycling, as can be seen in Figure 1g, when compared to that of cast-PEO SSLB.

For ZESSLBs with SPEs, the electrochemical cycling stability rapidly faded regardless of whether dry-PEO or cast-PEO was used, Figure 1i,j. Their Coulombic efficiencies for the first cycle were also significantly lower than those of all of the other cells at about 60%. At higher cycle numbers, both cells show increased concentration polarization during their discharge process, i.e., the plummeting of voltage in the discharge curves, indicating a continuous Li loss at the anode side, which cannot be shuttled back to the cathode. The capacity retentions of both cells after 20 cycles were only 25%, which is significantly lower than that of all the other cells, Figure 1k. The similarity of the low capacity retentions for both ZESSLBs with PEO SPEs would suggest that the side reactions initiated by the residual casting solvent are not the main cause of capacity loss in the ZESSLB setup. Furthermore, there is an irreversible areal capacity loss of about 0.06 mAh cm^–2^ in the first charging cycle for both ZESSLBs with PEO SPEs before the cell voltage reached 3.9 V vs Li/Li^+^, while this was not observed in the SSLB cells with PEO SPEs, i.e., comparing Figure 1i,j with Figure 1g,h. The irreversible capacity loss at the beginning of the cell charging process for ZESSLBs with PEO SPEs suggests that side reactions occurred once Li starts to deposit onto the Cu current collectors.

**Figure 2:**
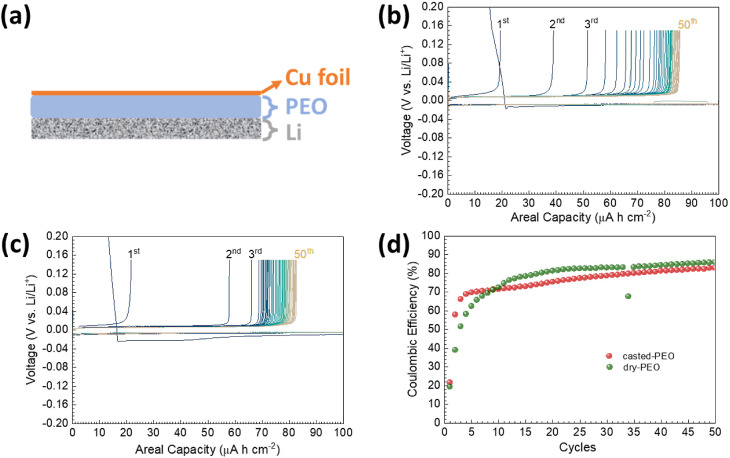
(a) Sketch of the zero-excess SPE symmetric cell. Electrochemical cycling of (b) dry-PEO zero-excess SPE symmetric cell, (c) cast-PEO zero-excess SPE symmetric cell, and (d) their respective cycling Coulombic efficiencies.

To exclude possible side reactions at the LLZO–PEO interface, dry-PEO and cast-PEO zero-excess SPE symmetric cells were made by using Li foils and Cu foil current collectors for electrochemical cycling, Figure 2a. For each cycle, 0.1 mAh cm^–2^, i.e., Li of about 0.5 μm thickness, was deposited onto the Cu foil and then stripped back to the Li foil until the cell polarization voltage reached 0.15 V vs Li/Li^+^, Figure 2b,c. An initial irreversible areal capacity of about 0.02 mAh cm^–2^ was observed for both cells prior to Li nucleation, which might be the result from an initial PEO decomposition to form an SEI and additionally reduce impurities, such as CuO, on the surface of the Cu foil. The Coulombic efficiency for the first cycle of both cells was as low as 20%, Figure 2d. For the zero-excess dry-PEO symmetric cell, the Coulombic efficiency increased gradually from cycle to cycle and reached about 86% after 50 cycles, while it improved much faster for the cast-PEO, from the first cycle at 20% to the second cycle at 58%, reaching 83% after 50 cycles. The difference in the Coulombic efficiency improvement upon cycling indicates that the SEI formation between dry-PEO and Li may be different from that between cast-PEO and Li. Furthermore, the very low Coulombic efficiencies, even after 50 electrochemical cycles, suggest a continuous loss of Li to either SEI formation or dead Li at the Cu current collector side. Since the assembled half-cells were purely solid, i.e., there was no free-flowing liquid, a continuous SEI formation would suggest that the decomposed products could offer an electrically conductive path through the SEI for electrons and ions to reach the interface of the SEI/Li.

**Figure 3:**
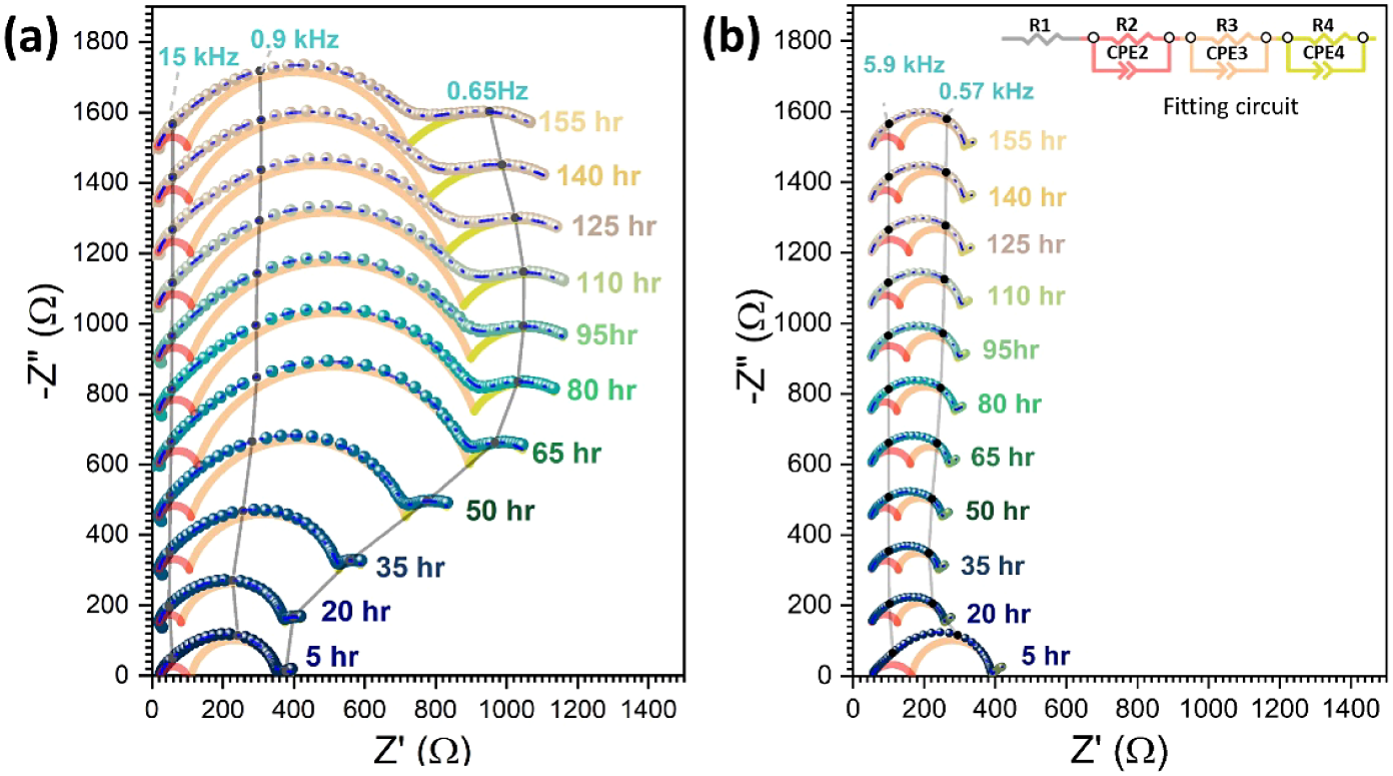
Time dependent EIS spectra of symmetric (a) Li/cast-PEO/Li cell and (b) Li/dry-PEO/Li cell with the corresponding fitting circuit Tsai inserted in Figure 3b. −*Z*” values were increased by 150 Ω per measurement on the −*Z*″ axis for better readability.

Symmetric Li/PEO/Li cells with dry-PEO and cast-PEO were assembled and held at 80 °C for time dependent electrochemical impedance spectroscopy (EIS) measurements, Figure 3. The measured data were analyzed by using an equivalent circuit, as presented in the inset of Figure 3b. At 80 °C, R1 can be assigned to the electrolyte resistance, R2/CPE2 and R3/CPE3 are assigned to contributions from the interfacial response because the fitting capacitances for CPE2 and CPE3 are in the range of 10^–7^–10^–6^ F cm^–2^ and R4/CPE4 is assigned to the diffusion of ions within the symmetric cells as to the fitting capacitance is in the range of 10^–2^–10^–3^ F cm^–2^.^35−37^

Calculated from R1, the total conductivity of cast-PEO was about 4.6 × 10^–4^ S cm^–1^ at 80 °C while that for dry-PEO was about 6.6 × 10^–4^ S cm^–1^ at 80 °C, which are comparable to previous studies showing values between 6 × 10^–5^ S cm^–1^ and 7 × 10^–4^ S cm^–1^.^11,18,38,39^ The depressed semicircle in the midfrequency range is related to the overall interfacial resistance as can be identified from the fitted capacitances. Appetecchi et al.^40^ and Li et al.^38^ suggest that R2/CPE2 or R3/CPE3 can be associated with the charge transfer resistance at the Li/SEI interface while the other one is associated with the growth of the passive layer. From Figure 3, it can be seen that R2/CPE2 semicircles remained almost constant upon increasing the dwell time while R3/CPE3 semicircles were changing. Therefore, R2/CPE2 can be assigned to the contribution from charge transfer resistance at the Li/SEI interface and R3/CPE3 can be assigned to the growth of the passivated SEI layer. Since the EIS measurements were only carried out with a perturbation voltage of 10 mV, the initial decrease of the R3 for Li/dry-PEO/Li symmetric cell from 5 to 20 h would not be possible to be the reducing of SEI thickness. It can only be attributed to the increase of electrical conductivity within the passivated SEI lay, as hypothesized by Liu et al. that lithium alkoxide formation from reduced ether bonds can increase the Li-ion transport.^41^ When the time dependent EIS for cast-PEO is compared with that for dry-PEO, the reactivity between cast-PEO and Li is much higher than that between dry-PEO and Li. This agrees with the observations from Appetecchi et al.^42^ and Shin et al.^37^ who suggest that the residual solvent reacts with Li metal more pronouncedly, increasing the passivated SEI resistance as well as the cell impedance. Therefore, the measured R3 could be a competition result between SEI growth (increase of R3) and the increase of electrical conductivity (decrease of R3) of SEI. However, we can not exclude the initial decrease of Li/PEO/Li symmetric cells due to the improvement of the interfacial contact between Li and PEO. Interestingly, R4 was continuously increasing throughout the measured time for the cast-PEO cell (while it was difficult to determine for the dry-PEO cell due to spur like data hindering a precise fitting). This indicates that the ions experience a larger diffusion barrier within the SPE with the increase of time, while the measured SPE conductivity R1 was similar. Moreover, the resistance of the passive SEI layer (R3) decreased after 95 h, while the diffusion of ions within the SPE (R4) was further impeded. The almost unchanged R1 and R2 indicated that the impeding of ion transfer within the Li/cast-PEO/Li symmetric cell would be majorly within the passivated SEI. As the charge transfer phenomenon in the SEI is affected by its chemical composition, the continuous change of R3 and R4 indicates the incessant reactions between the chemical compositions within the SEI as well as those with PEO and/or Li.

**Figure 4:**
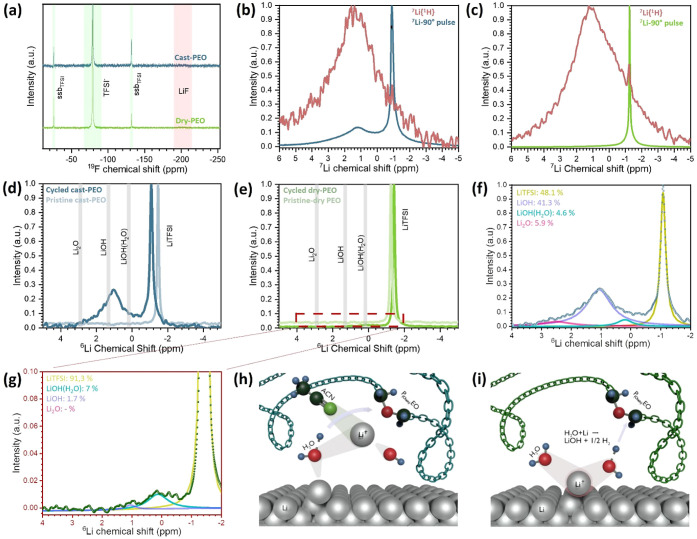
Solid-state MAS NMR spectra of cycled PEO samples from ZESSLBs. Intensities are normalized. (a) ^19^F NMR of cycled PEO samples. ^7^Li single-scan and ^7^Li{^1^H} cross-polarization of cycled (b) cast-PEO and (c) dry-PEO. ^6^Li NMR of (d) cast-PEO and (e) dry-PEO with their corresponding fitting data as a percentage of the total integrated area of (f) cast-PEO and (g) dry-PEO. Sketches of possible reactions with Li for (h) both residual water and ACN and (i) only water.

Solid-state NMR spectroscopy was used to study the composition of the Li/PEO reaction products, Figure 4. The cycled ZESSLBs with PEO SPE were discharged to 3.4 V to shuttle the active Li in the anode back to the cathode. Afterward, the PEO samples were taken off from the ZESSLBs for NMR studies. In the ^19^F-NMR spectra, only the TFSI^–^ peak and its corresponding spinning sidebands equidistant at higher and lower chemical shifts were observed for both cycled dry-PEO and cast-PEO, Figure 4a. There is no indication of other fluorine species, such as LiF, which is expected to occur around 204.3 ppm.^43^ This indicates that the TFSI^–^ anion is relatively stable during the electrochemical cycling. Therefore, no significant difference in LiTFSI concentration in PEO SPE between cycled and pristine samples can be expected.

^7^Li solid-state NMR spectra of both the cycled cast-PEO, Figure 4b, and the cycled dry-PEO, Figure 4c, exhibit a narrow signal at around −1.1 ppm, which is assigned to LiTFSI.^44^ Additional broad resonance signals were detected at a higher chemical shift, with a maximum intensity at around 2 ppm. This broad signal indicates the presence of additional solid phases, which typically exhibit comparably short spin–spin relaxation times and, thus, broad NMR signals.

To further investigate the solid phases, we conducted ^7^Li{^1^H} cross-polarization experiments. As for single-pulse experiments, ^7^Li{^1^H} spectra exhibit resonances in the ^7^Li chemical shift dimension. However, the signal originating from the ^7^Li nuclei in close proximity to the ^1^H (∼a few angstroms) nuclei is enhanced through the transfer of magnetization. The acquired cross-polarization spectra exhibiting resonances centered around 2 ppm indicate that these resonances result from a ^1^H-containing solid phase, likely LiOH and/or LiOH·(H_2_O)_*x*_ where ^7^Li–^1^H distances are only a few angstroms facilitating cross-polarization. Additionally, ACN may substitute H_2_O as ligand-bound crystal water, resulting in multiple new detectable species. Comparing the ^7^Li{^1^H} spectra of cycled cast-PEO and cycled dry-PEO, the signals’ position and line width are very similar.

To increase the spectral resolution, ^6^Li NMR measurements of the cycled and the pristine PEO samples were acquired, Figure 4d,e. The ^6^Li NMR spectrum of the pristine cast-PEO shows one signal at around −1.5 ppm, which can be attributed to LiTFSI, Figure 4d. After electrochemical cycles, the LiTFSI signal of cycled cast-PEO appears at −1.1 ppm while another significant signal appears at 1.1 ppm, which could be assigned to LiOH (see Huff et al.^45^ and Meyer et al.^46^) However, susceptibility effects in the sample, which could be the reason for the chemical shift difference of LiTFSI, could also hint at LiOH·(H_2_O) or LiOH·(H_2_O)_*x*_ phases. As previously discussed, this broad signal could also be a superposition of multiple side products, such as the aforementioned LiOH with differing ratios of ligand bound water and ACN.

LiOH and its crystal water analogues could form through the reaction of metallic Li and residual water, which was tightly bound into the polymeric void volume. It is also possible that the LiOH is a side reaction product of the PEO degradation when the carbon species of PEO produced aromatic compounds as suggested by Ushakova et al.^29^ These side reactions could release hydrogen and oxygen atoms from the polymer, which might further react with water. DFT modeling by Mirsakiyeva et al.^30^ suggested that Li_2_O could be a possible reaction product from the reaction between PEO and Li. This matches resonances at around 2.8 ppm in cycled cast-PEO. In contrast, resonances indicating Li_2_O were not identified in the spectrum of cycled dry-PEO.

Peak fitting was used to deconvolute the ^6^Li NMR spectra of cycled cast-PEO and dry-PEO to approximately quantify side reaction products, Figure 4f,g. We used four Lorentzian lines assigned to LiTFSI (∼1.8 ppm), LiOH·(H_2_O) (∼1.3 ppm), LiOH (∼0.3 ppm), and Li_2_O (∼2.8 ppm) for the ^6^Li NMR spectrum of cycled cast-PEO, Figure 4f. The spectrum of the cycled dry-PEO sample with much lower intensity was fitted using three Lorentzians contributions approximately representing LiTFSI, LiOH·(H_2_O), and LiOH due to the chemical shift where Li_2_O would resonate a signal was not possible to be distinguished from the background noise, Figure 4g.

The capacity lost to side reactions at the anode may be estimated by considering the integral of the Lorentzian line fits. Usually, the integral in the NMR spectra is proportional to the number of resonating ^6^Li nuclei, when using a sufficiently large delay between scans allowing for spin–lattice relaxation. To estimate the amount of side products, we used the LiTFSI peak integral as a reference, assuming an equal LiTFSI concentration in the samples. This is based on an identical initial concentration and the absence of side-product indications in the ^19^F NMR spectra. The amount of LiTFSI (*n*_LiTFSI_) was calculated by multiplying the concentration and volume of the PEO electrolytes. Using *n*_LiTFSI_, the amount of Li lost upon cycling can be roughly quantified using fitted peak integrals ∫[Peak_Li-lost_]. The capacity lost to the reaction between SPE and Li, C_loss_, can be estimated by
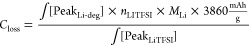
where ∫[Peak_Li-lost_] denotes the area fraction of non-LiTFSI peaks, ∫[Peak_LiTFSI_] denotes the areal fraction of the LiTFSI peak, *n*_LiTFSI_ denotes the molar quantity of LiTFSI in the PEO SPE of the cells (1.4 × 10^–5^ mol for cast-PEO, 4.9 × 10^–5^ mol for dry-PEO), *M*_Li_ is the molar mass of Li, and 3860 mAh g^–1^ is the theoretical gravimetric capacity of Li metal. The estimated capacity loss through the formation of side reaction products for the cell containing cast-PEO is roughly 0.4 mAh cm^–2^, while that for dry-PEO is only roughly 0.1 mAh cm^–2^. While the absolute quantity is based on several assumptions, relative comparisons between cast-PEO and dry-PEO can be drawn based on this outcome. As we control the active material loading in the cathode for every cell to be similar, the first discharge capacity for all the cells in Figure 1 can be expected to be ∼0.8 mAh cm^–2^. By a comparison of the expected capacity for the first discharge, i.e., ∼0.8 mAh cm^–2^, with the 20th discharged capacity, i.e., ∼0.2 mAh cm^–2^, there was about 0.6 mAh cm^–2^ of capacity lost for both PEO ZESSLBs, Figure 1i,j. It shows that there was additional Li loss that could not be accounted for by the calculated loss capacitances from the measured NMR spectroscopy.

The difference between the capacity losses calculated from NMR spectra for cast-PEO and dry-PEO provides evidence for the residual ACN playing a major role in the formation of the side reaction products. Pons et al.^47^ and Rupich et al.^48^ suggest that the reaction of Li and residual ACN in PEO produces methane and lithium cyanide (LiCN). LiCN can further degrade into LiOH and hydrogen cyanide (HCN) if it is in contact with water. LiCN can also diffuse as organolithium salts into the bulk PEO where it reacts again with the nondepleted residual water, which is bound to the free volume of PEO to form more LiOH and LiOH·(H_2_O).^49^ Nevertheless, it is also possible that ACN as a stronger polarizing solvent than water leads to a solvent exchange with previously crystal bound water in LiOH·(H_2_O) to free it up directly on the interface to further react with Li. The difference would be a more stable LiOH·(H_2_O) layer in dry-PEO, Figure 4h, and a less stable LiOH·(H_2_O/ACN) layer in cast-PEO due to the formation of organolithium, which can conduct ions, Figure 4i.^41^

**Figure 5:**
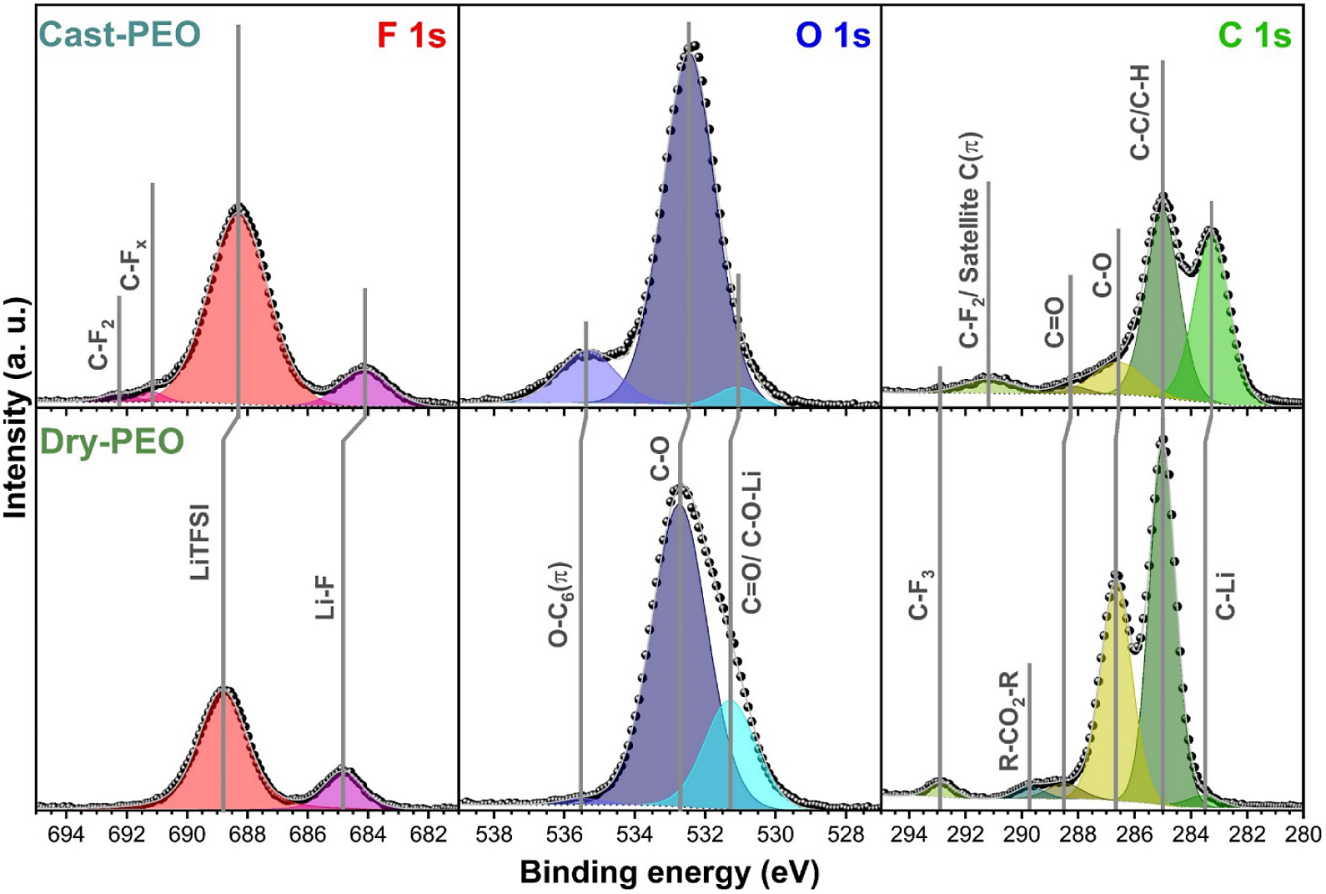
F 1s, O 1s, and C 1s spectra from XPS measurements of the cycled cast-PEO and cycled dry-PEO. The C–C/C–H signal was calibrated to 285 eV. The spectral intensity of each scan is normalized to the respective highest peak (C–O in cast-PEO; C–C/C-H peak in dry-PEO). The black points are the measured data, and the gray line indicates the accumulated fitting results.

Figure 5 shows the F 1s, O 1s, and C 1s XPS spectra of cycled cast-PEO and cycled dry-PEO. It is worth noting that the binding energies between cycled cast-PEO and cycled dry-PEO exhibit shifts up to 1.8 eV for specific species, which could be attributed to differing charging effects due to the differing electronically insulating properties of PEO and the formed SEI species.^50−52^

In the F 1s spectra of cycled cast-PEO and cycled dry-PEO, two major chemical environments were identified. Since the only source of fluorine is LiTFSI, the major peak at ∼689 eV can be attributed to the C–F_3_ moiety of LiTFSI while the peak at ∼684.5 eV indicates the presence of LiF.^32,33,53^ The detection of LiF implies the decomposition of LiTFSI when in contact with metallic Li. Nevertheless, LiF was not detected by NMR. This indicates that LiF should only be formed in a very small quantity and may exist only at the interface of Li and PEO where LiTFSI is in direct contact with metallic Li. For the cycled cast-PEO, it is notable that another two small peaks at higher binding energies, i.e., at ∼691 and ∼692 eV, were detected. These peaks at high binding energy are explained by Guéguen et al.^54^ as possible experimental artifacts related to the local charging effect of the electronically insulating PEO during XPS analysis. Nevertheless, similar detection was reported by Farhat et al.^55^ and Eshetu et al.^56^ for their cycled cell without any explanation for the observation. Yet, the binding energy peaks higher than the fluorinated electrolyte salts were assigned to C–F_2_ and C–F_*x*_ bonds according to previous studies.^57−60^ Therefore, it is very likely that the observed peaks are C–F_2_ and C–F_*x*_ species that formed from the long-term electrochemical cycling of LiTFSI, undergoing continuous decomposition in an electric field.

The analysis of O 1s XPS spectra is rather complicated due to the overlap of carbon–oxygen binding energies from PEO and the ones of lithium carbonate, lithium hydroxide, lithium oxide, and lithium peroxide.^29,32,33,52,61^ Usually, the most intense peak at ∼533 eV is attributed to C–O single bonds of the ether links, while the assignment of C=O double bonds from PEO ether links or the lithium alkoxide species, C–O–Li, is at ∼531 eV. Furthermore, an extra peak at ∼535.5 eV was identified in the O 1s XPS spectra, which can be attributed to the ether oxygens^62^ or oxygen close to a benzene ring from the cross-linking benzophenone, i.e., O–C6(π).^63^ The detection of such high relative intensity bonds in cast-PEO but very weak ones in dry-PEO favors the O–C6(π) bond, indicating better PEO cross-linking with benzophenone. As both PEO and benzophenone are dissolved in ACN for mixing in molecular level, the homogeneity of benzophenone in cast-PEO would be much higher than that in dry-PEO. Furthermore, with the residual ACN in the SPE having a plasticizing effect, this could increase the benzophenone molecular diffusion in the polymer.^64^ Less crystallinity in the SPE and better diffusion of the benzophenone between the PEO chains could thus increase the likelihood of activated benzophenone radicals reacting with the polymer chain during the cross-linking process. Furthermore, it is noticed that the intensity ratio of C–O/C=O is much higher for the cycled cast-PEO than that for the cycled dry-PEO. Considering the results from the NMR studies that a much higher amount of LiOH was detected in the cycled cast-PEO, it is reasonable to conclude that LiOH (∼532 eV) significantly contributes to the intensity of the C–O signal while blocking the C=O signal in the O 1s XPS data of cycled cast-PEO.

The most intense peak of the C 1s spectra from both samples is at ∼285 eV, which is caused by single C–C and C–H bonds from PEO.^29,32,33^ The ∼286.5 eV binding energy peak can be assigned to the ethereal C–O bonds from PEO. When using C–C/C-H peak intensities as references, the relative intensity of detected C–O bonds in the cycled cast-PEO is less than that in the cycled dry-PEO. The observation supports the conclusion from O 1s spectra that LiOH contributes to the detection of C–O signal at ∼532.5 eV for the cycled cast-PEO if one would consider C–O bonds are only attributed from PEO, which should be expected to be equal for both samples. Furthermore, the detection of C–F_2_ species from the cycled cast-PEO at 291 eV supports the hypothesis that long-term electrochemical cycling of the battery causes LiTFSI to undergo a continuous decomposition to form C–F_2_. The most pronounced difference in the C 1s spectra between cycled cast-PEO and cycled dry-PEO is the scale of the detected C–Li peak at 283.5 eV, which indicates PEO decomposition when in contact with Li. The higher relative intensity of C–Li bonds in cycled cast-PEO can be explained by the reaction between residual acetonitrile and deposited Li to form alkyl-lithium species. The highly reactive alkyl-lithium can further react with the residual water in PEO to form LiOH and attacks PEO by bond cleavage of the oxygen ether species.^48,65,66^ The bond cleavage process would then yield additional Li alkoxides by consuming additional Li. The heightened organolithium increases the electrical conductivity of the SEI, as the radical species formed during these processes can be electrically conductive.^29,67^ The nonnegligible electrical conductivity would then allow for the continuous growth of the SEI. This explains the observation of a continuous growth of the SEI layer from the EIS measurement and the higher concentration detection of LiOH from the NMR measurement of the cast-PEO sample.

**Figure 6:**
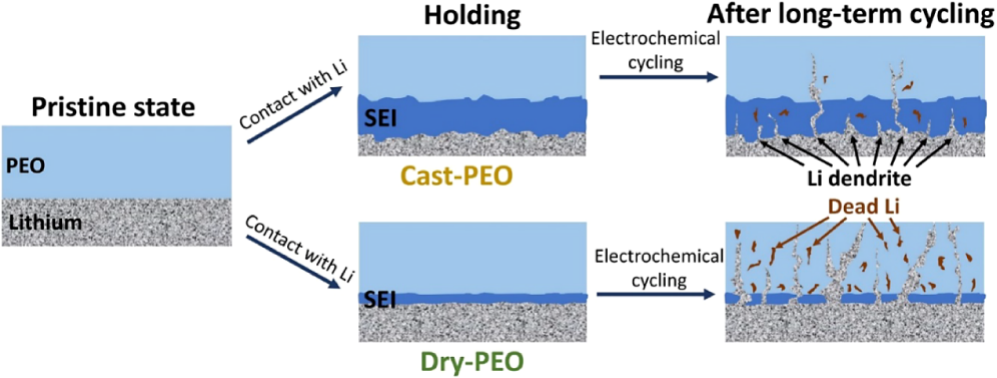
Schematic drawing of the SEI and dead Li formation in PEO when cast-PEO and dry-PEO are electrochemically cycled in direct contact with metallic Li.

Based on the experimental results from ZESSLBs and Li/PEO/Cu symmetric-cells, the capacity loss upon electrochemical cycling of both cells using dry-PEO and cast-PEO is similar, i.e., the capacity retention from ZESSLB and the low Coulombic efficiencies of the symmetric cells. However, EIS and NMR results suggest that the reaction between the cast-PEO and Li is much higher than that for the dry-PEO and Li, i.e., a thicker SEI was detected in the cycled cast-PEO than that in the cycled dry-PEO. A reasonable explanation for the low Coulombic efficiencies for ZESSLBs and Li/PEO/Cu symmetric cells could be a higher portion of deposited Li forming isolated Li within the cycled dry-PEO while more of the deposited Li was consumed to form SEI for the cycled cast-PEO during electrochemical cycling, Figure 6. Therefore, a dramatic capacity loss was not observed when a Li reservoir was used in SSLBs, i.e., Figure 1f,g. The easier formation of isolated Li in the dry-PEO system could be the result of a thinner SEI compared to the one formed in the case of cast-PEO, which also implies easier Li dendrite growth for the dry-PEO than that for the casted-PEO due to the casted-PEO having better SEI protection. Nevertheless, both the dry-PEO and cast-PEO are facing significant Li loss when directly in contact with Li in the ZESSLB setup, which withdraws the suitability of using PEO as the interlayer for ZESSLB application. Furthermore, a protective interlayer that prompts homogeneous Li deposition and separates deposited Li from PEO SPE, such as composite, might be beneficial when using PEO as the backbone solid electrolyte for ZESSLB fabrication.^68^

## Conclusion

PEO SPEs were examined for their ZESSLB applications using all-ceramic half-cells. The electrochemical cycling tests demonstrated that the ZESSLB with PEO SPEs experienced fast capacity fading when compared to SSLB with PEO SPE. Li/PEO/Cu symmetric cells show that both dry-PEO and cast-PEO have very low average Coulombic efficiencies for only about 86%, which indicates a dramatic Li loss during electrochemical cycling. EIS measurements suggest that the cast-PEO formed a much thicker SEI than that for the dry-PEO. NMR measurements reveal that the SEI are mostly composed by LiOH, LiOH·(H_2_O), and Li_2_O while XPS suggests that LiTFSI can decompose at the interface between PEO and deposited Li. As the detection of C–Li bonds by XPS indicates the decomposition of PEO, the much higher concentration of C–Li bonds in cast-PEO suggests that the residual acetonitrile can react to lithium to form alkyllithium species to further decompose PEO to form lithium alkoxide and organolithium, which may provide electrical conductivity for continuous SEI formation for the cast-PEO. When considering the similar capacity fading for both ZESSLBs and the different degrees of SEI formations by using cast-PEO and dry-PEO, a higher degree of isolated lithium formation within dry-PEO than cast-PEO could be the reasonable explanation. Overall, the use of a PEO solid electrolyte as the backbone for ZESSLB fabrication would only be possible when PEO and lithium are not in direct contact, implying a suitable interlayer to avoid direct contact is necessary.

## Experimental Section

The composite LCO/LLZO–LLZO half-cells were prepared according to our previous publications.^34,69^ The all-ceramic LCO/LLZO–LLZO half-cell fabrication was done by sintering an LCO/LLZO composite positive electrode (CPE) onto LLZO discs. The CPE was prepared using a 1:1 mass ratio of LCO (MTI Corp., USA), 5.06 g cm^–3^, and LLZO, 5.35 g cm^–3^, powders. The powders were weighed and milled by using yttrium stabilized zirconia balls, and pure ethanol was used as a solvent for 24 h to reduce the particle size, as in ref (69). The slurry was further collected and dried at 60 °C in a vacuum oven. Then, the screen printing ink was prepared by using a three-roll mill (Exakt 50, Germany) to mix the slurry with a weight ratio of 3 wt % ethyl cellulose (Sigma-Aldrich) in terpineol (Sigma-Aldrich): solid loading of 1:1. Afterward, the ink was painted onto the LLZO discs with a brush and dried at 55 °C in air several times to reach the desired green ink loading. The cells were sintered in a tube furnace (Nabertherm, Germany) with a heating rate of 6.5 K min^–1^ to 970 °C with 15 min of dwell time using an Al_2_O_3_ ceramic boat as a carrier in air. Then, free cooling was applied to the furnace for its temperature to drop to RT. The LCO loading on a cell was calculated by the difference in weight between the used LLZO disc and that for the cell after the sintering. Typical cells used in this paper have CPE loadings ∼14 mg cm^–2^ to give LCO loading ∼7 mg cm^–2^.

PEO (Sigma Aldrich, USA, 4 × 10^5^ g mol^–1^) was dried at 50 Pa of vacuum and 80 °C for 2 days to remove residual water. All consecutive steps of the PEO preparation were conducted in a glovebox (MBraun, O_2_ < 0.1 ppm, H_2_O < 0.1 ppm). For the fabrication of the cast-PEO, 0.28 g PEO was mixed with 0.1216 g LiTFSI (IoLiTec, Germany) for an ethylene oxide repeating unit to a LiTFSI ratio of 15:1 and 0.014 g benzophenone (Merck, Germany) as the cross-linking agent. The mixture was stirred overnight in 12 g of anhydrous ACN (VWR, France). The solution was cast in a Teflon Petri dish to form a film. The solution was first kept under 950 mbar for 1 day and flushed with argon every 2 h. For the next 3 days, the vacuum was stepwise increased to 900, 800, 500, and 10 mbar. Then, the film was kept under 10^–2^ mbar for another 24 h before it was heat treated under normal pressure at 100 °C for another 5 h. An UVAcube100 UV oven (Dr. Höhnle AG, Germany) was used for UV induced cross-linking of the PEO chains via the benzophenone. Dry-PEO was prepared with the same ratio of the precursors, without the use of any ACN.^15,24^ In short, for fabricating dry-PEO, 0.28 g of properly dried PEO and 0.014 g of benzophenone were mixed first using a mortar and pestle by hand thoroughly. Then, 0.1216 g of properly dried LiTFSI was added slowly into the mixture with grinding. It is worth mentioning that once LiTFSI is added into PEO, the PEO powder starts to form a dough- or rubber-like bulk material. After LiTFSI was completely added into PEO, the “dough” was sandwiched between two Mylar foils and hot-pressed at 100 °C for several minutes to form a thick film. The film was taken out from Mylar foils and folded symmetrically 2 times to put back to Mylar foils for another hot pressing. The folding and pressing of the processes were repeated about 15 times before it can be used for battery assembly. All of the processes for dry-PEO preparation were carried out inside a dry room.

For battery cell assemblies, the sintered LCO/LLZO–LLZO half-cells were polished on the LLZO side to remove possible impurities and thin the solid electrolyte down to ∼400 μm using SiC sandpapers. Au thin film was sputtered onto the CPE surface (Cressington 108 auto coater, UK) to serve as a current collector. For SSLB, Au thin film was sputtered onto the LLZO surface to help Li adhesion and heated to 250 °C to increase the bonding between the LLZO and Li anode on a hot plate before it was put into Swagelok cells for electrochemical tests. For all-ceramic ZESSLB, an Au thin film was also sputtered onto LLZO surface to help Li deposition during the charge process. Six micrometer thick Cu foil was attached onto the LLZO by using isostatic press for 30 s under a pressure of 500 MPa. For the cells with PEO solid electrolytes, the PEO films were attached onto the bare LLZO surface of the LCO/LLZO–LLZO half-cell directly by hand. Either Li or Cu foil was attached also by hand onto the PEO film to serve as the anode for SSLB or the current collector for ZESSLB. The cells were sandwiched between two Ni plates in Swagelok cells. All the Swagelok cells were using springs with a spring constant k = 10 N cm^–1^ for providing pressure and contacts to the rods for electrical connection. All the battery cell assemblies were conducted inside a glovebox under an argon atmosphere with O_2_ < 0.1 ppm and H_2_O < 0.1 ppm. Stainless steel Swagelok cells were used as cell housing for all the electrochemical tests. Electrochemical cycling was conducted using a VMP3 potentiostat (Biologic, France) in an 80 °C climate chamber (Binder GmbH, Germany). The cells for the electrochemical cycling were equilibrated for 3 days before electrochemical cycling began. The cells were charged at a constant current density of 50 μA cm^–2^ until they reached a potential of 4.2 V where the voltage was held until the charging current density dropped to 10 μA cm^–2^. The cells were discharged with a current density of 50 μA cm^–2^ until the cell voltage reached 3.4 V. Cu-PEO-Li half-cells were charged and discharged with a current density of 20 μA cm^–2^. Charging was limited to 5 h, while the discharge was finished when the voltage reached 0.15 V. EIS measurements for Li/SPE/Li symmetric-cells were performed with a perturbation voltage of 10 mV in the frequency range from 1 MHz to 0.1 Hz. Measurements were conducted by using two 1 cm^2^ surface area Li electrodes, which were separated by a 12 mm diameter dry-PEO or cast-PEO membrane.

## NMR-Spectroscopy

Samples for NMR were extracted by removing cross sections of the PEO layer from the ZESSLBs, which have been subjected to 20 charge–discharge cycles, ending on a full discharge to 3.4 V. All solid-state NMR measurements were performed in a 3.2 mm triple resonance H/X/Y CPMAS probe head at a constant temperature of 25 °C. ^19^F MAS NMR spectra were acquired on a Bruker Avance III HD instrument within a 9.4 T magnet. Data acquisition was performed via a rotor synchronized Hahn-echo pulse sequence, using 2.8 and 5.6 μs for the 90° and 180° pulses. Samples were spun at 20 kHz MAS spinning speed, accumulating 128 scans using a relaxation delay of 10 s between scans. ^6^Li and^7^Li MAS NMR spectra were acquired on a Bruker AvanceNEO instrument within an 18.8 T magnet. The MAS spinning speed was 12 kHz. ^6^Li spectra were recorded following a 4.5 μs single-pulse, accumulating 8192 scans using a relaxation delay of 5s. ^7^Li spectra were recorded following a 6.6 μs pulse, accumulating 512 scans using a relaxation delay of 5s. Rotor-synchronized ^7^Li cross-polarization was performed by using a MAS spinning speed of 24 kHz. Following a 5.25 μs ^1^H single-pulse excitation, cross-polarization occurred during a contact time of 3 ms. Subsequent ^7^Li signal acquisition was accompanied by proton decoupling. 512 scans were accumulated using a relaxation delay of 5s.

## X-Ray Photoelectron Spectroscopy

X-ray photoelectron spectroscopy was conducted on a Kα spectrometer connected to a glovebox (Thermo Fisher, USA), enabling measurements without air contamination. The instrument has an Al–Kα X-ray source and was operated at a 10^–9^ mbar base pressure. The measurements were conducted with a pass energy of 50 eV and a spot size of 400 μm on the sample. The survey spectra were obtained using a pass energy of 200 eV and the same spot size as those of the elements. The samples were evaluated using the software Avantage (Thermo Fisher).
